# Change over time in characteristics and survival of residents newly admitted to nursing homes: an analysis of health insurance claims data from 2011 to 2020 in Germany

**DOI:** 10.1007/s10433-025-00871-z

**Published:** 2025-07-21

**Authors:** Hannes Jacobs, Stephanie Stiel, Anna Völkel, Tanja Schleef, Birte Burger, Jona Theodor Stahmeyer, Kathrin Wandscher, Anna Levke Brütt, Falk Hoffmann

**Affiliations:** 1 Department of Health Services Research, Carl Von Ossietzky Universität Oldenburg, Oldenburg, Germany; 2 Institute for General Practice and Palliative Care, Hannover Medical School (MHH), Hannover, Germany; 3 Health Services Research Unit, AOK Niedersachsen, Hannover, Germany; 4 Department of Medical Psychology, University Medical Center Hamburg-Eppendorf, Hamburg, Germany

**Keywords:** Long-term care, Time trends, Survival, Mortality, Dementia, Cancer

## Abstract

**Background:**

Studies on changes in characteristics and survival of nursing home residents (NHRs) are rare. Therefore, this study aims to analyze ten-year trends in newly admitted German NHRs.

**Methods:**

For this retrospective cohort study, claims data of a regional German health insurance fund was used to identify all residents aged 65 years and older newly admitted to a nursing home between 2011 and 2020 (with a follow-up period ending on December 31, 2021). Characteristics of NHRs were analysed descriptively and stratified by 5 two-year cohorts. Survival times and mortality were assessed by applying the Kaplan–Meier-method and a cox regression was used.

**Results:**

A total of 113,929 residents were newly admitted between 2011 and 2020 (69% female; mean age 83.8 years). Over the years, the proportion of men and the mean age slightly increased. Prevalence of dementia remained nearly stable (45–48%) while cancer prevalence raised from 30 to 37%. Overall, median survival time decreased from 745 days in 2011/2012 to 615 days in 2019/2020. Survival times in residents with dementia decreased significantly (median survival from 790 to 651 days) while it remained nearly unchanged in cancer patients (from 444 to 410 days).

**Conclusions:**

We found that survival after nursing home admission decreased in Germany from 2011 to 2020, which was accompanied by shorter survival in residents with dementia and by an increasing proportion of those with cancer, who already experience higher mortality. There is a growing need to integrate palliative care in nursing homes and to also provide appropriate care for older patients with cancer outside nursing homes.

**Supplementary Information:**

The online version contains supplementary material available at 10.1007/s10433-025-00871-z.

## Introduction

The aging population in most industrialized countries and the increasing life expectancy are leading to challenges for healthcare systems (The Lancet Healthy Longevity 2022). In the European Union, for example, it is estimated that 38.1 million people are projected to require long-term care (LTC) in 2050, an increase of 24% compared to 2019 (European Commission 2022). Despite policy efforts to expand home-based care and promote “ageing in place” (OECD 2018; Pani-Harreman et al. 2021), the number of individuals residing in nursing homes remains substantial and is expected to grow further. This trend reflects the increasing number of people with complex care needs (Marengoni et al. 2011; European Commission 2021) and underscores accompanied persistent challenges faced by health systems worldwide, including financial sustainability, health inequalities and unequal access to high-quality care (GBD 2018; OECD 2023).

Nursing home residents (NHRs) are often characterized as being female as well as being affected by multiple chronic diseases and associated physical and cognitive limitations (Auer et al. 2018; Lane et al. 2019; Ng et al. 2020; Pacifico et al. 2023; Gjøra et al. 2024; Czwikla et al. 2024). Dementia, in particular, is a leading cause of nursing home admission (Luppa et al. 2010; Sköldunger et al. 2019), as it eventually necessitates continuous care and supervision. Post admission, due to its gradual progression dementia is associated with longer lengths of stay (Collingridge Moore et al. 2020). Prevalences in nursing homes across European countries range from 55 to 85%, making dementia a central concern in this population (Auer et al. 2018; GjØra et al. 2021; Pacifico et al. 2023; Czwikla et al. 2024). In Canada, a study observed that the prevalence among newly admitted NHRs increased from 42% in 2000 to 54% in 2015 (Ng et al. 2020). However, time trends for Europe are barely investigated (Brück et al. 2025). On the contrary, cancer is with a prevalence ranging between 8 and 20% also common in European NHRs (Liuu et al. 2019; Villani et al. 2022; Berloge et al. 2024) but typically linked to shorter, more intensive terminal phases, frequently leading to shorter lengths of stay (Collingridge Moore et al. 2020). Furthermore, the English Longitudinal Study of Ageing (ELSA) indicates an increasing trend in the prevalence of cancer (Green et al. 2017). However, high-quality studies on changes in prevalence over time are clearly lacking here as well.

Parallel to this, end-of-life care is increasingly shifting to nursing homes (Forma et al. 2017; Aaltonen et al. 2017), which are becoming an ever more prominent setting for the last phase of life and dying (Stiel et al. 2023). Data on mortality and survival following nursing home admission vary across countries, with most studies reporting a mortality within 12 months ranging between 17 and 42% (Hjaltadáttir et al. 2011; Tabue-Teguo et al. 2015; Vetrano et al. 2018; Vossius et al. 2018; Reilev et al. 2019; Collingridge Moore et al. 2020; MacRae et al. 2024). Median survival times vary from 705 days in Germany (Allers and Hoffmann 2018; Hoffmann and Allers 2019) to 930 days in Iceland (McCann et al. 2009; Wieland et al. 2010; Hjaltadáttir et al. 2011; Li et al. 2018; Vossius et al. 2018). However, only few studies have investigated time trends in survival after nursing home admission. Swedish data suggest a decline in median survival after institutionalization from 764 days in 2006 to 595 days in 2012 (Schön et al. 2016). Hoben et al. also showed decreasing median survival times for two Canadian regions, while for another region, an increase was observed (Hoben et al. 2019). Furthermore, data from Iceland for 1996 to 2003 indicate a general decrease of survival over time but without following a clear pattern (Hjaltadáttir et al. 2011). Overall, most studies are rather old or inconclusive. Moreover, as mortality is highest soon after institutionalization (Schön et al. 2016; Allers and Hoffmann 2018; Braggion et al. 2020; Jorissen et al. 2024), investigating short-term survival is also important, but most existing studies focused on long-term outcomes (median survival time, mortality within at least 12 months).

Consequently, this study aims to investigate time trends in both the characteristics of newly admitted NHRs in Germany as well as survival times and mortality. Furthermore, special attention is given to short-term periods after institutionalization and to NHRs with dementia and cancer.

This study contributes to the literature in several ways. On the one hand, time trends in characteristics of newly admitted NHRs and their survival after nursing home admission are barely investigated, although a deeper understanding of how resident characteristics and survival patterns at nursing home admission have evolved over time seems crucial for anticipating future demands on LTC systems. On the other hand, dementia and cancer are very prevalent diagnoses in nursing homes and associated with markedly different trajectories of care needs and end-of-life processes and therefore, highlight the complexity of care provision in nursing homes by being sensitive to heterogenous needs of different population groups. Thus, we also provide a valuable contribution to help health care policies to reduce health and care inequalities between different groups.

## Study context

Germany provides a well-suited context for analysing trends in the characteristics of NHRs and their survival following institutionalisation. Since the introduction of mandatory LTC insurance in 1995, access to publicly funded LTC has been based on standardised eligibility assessments done by an independent institution (Medizinische Dienst) (Bundesministerium für Gesundheit 2019; Wenke et al. 2021). Between 1995 and 2016, eligibility was determined by assigning individuals to one of three care levels, which were based on the time required for basic care. Care level I required a minimum of 90 min of care per day (of which at least 45 min had to be for basic care, e.g., bathing, dressing, eating), level II required 180 min (with 120 for basic care), and level III required 300 min (with 240 for basic care) (GKV-Spitzenverband 2024). In order to provide a more comprehensive assessment of the individual's level of independence and competencies in the context of physical, cognitive, or psychological impairments, these levels were modified into five care grades in 2017 (Nadash et al. 2018). Grade 1 reflects minor impairments, while grade 5 reflects the most severe impairments with extensive care needs (GKV-Spitzenverband 2024). Eligibility for institutional care typically begins at grade 2. For more information on German LTC and the overall health care system see Blümel et al. (2020).

Access to institutional LTC varies substantially across countries due to differences in financing mechanisms, eligibility criteria, and policy priorities. In Germany, between 2010 and 2021, the number of individuals receiving institutional care rose from approximately 700,000 to over 900,000 (DESTATIS 2025a). In 2023, 69% of NHRs were female, and 94% were aged 65 years or older (73% were 80 years or older), marking a substantial increase since 1999 (65 years or older: 91%; 80 years or older: 66%). In addition, 17% had a care grade 1 or 2, 69% 3 or 4 and 14% grade 5 (DESTATIS 2025b). Despite universal access, out-of-pocket contributions for institutional LTC remain high by international comparison. Public funding covers approximately 64% of total LTC costs, compared to 80–90% in countries like the Netherlands and Norway (OECD 2020). In 2021, Germany spent 2.2% of its gross domestic product on LTC—below countries like the Netherlands (4.1%) or Sweden (3.2%), but above the OECD average of 1.7% (OECD 2020). Given the projected demographic shift—with the share of people aged 65 and over expected to rise from 22% in 2022 to nearly 29% by 2050 (DESTATIS 2025c)— further cost increases are anticipated.

## Material and methods

### Study design and study population

This retrospective study was conducted as part of the *Gut-Leben* project, which aims to evaluate the implementation of and barriers for advance care planning (ACP) in German nursing homes (Stiel et al. 2023). The analyses were based on anonymised claims data obtained from the AOK Niedersachsen, a large statutory health and LTC insurance fund. The AOK Niedersachsen represents approximately 3.1 million members, which corresponds to 38% of the population in Lower Saxony, one of Germany's largest federal states with 8.1 million inhabitants (AOK Niedersachsen 2025).

We included a cohort of all individuals who were newly admitted to a nursing home between January 1, 2011, and December 31, 2020 (with a follow-up period ending on December 31, 2021), and who had been continuously insured for at least 365 days without prior nursing home placement. Moreover, individuals had to be at least 65 years of age at the beginning of each year.

### Data collection

In order to evaluate characteristics of the newly admitted nursing home population, information on sex, year of birth, care dependency, and outpatient diagnoses were obtained from claims data. Furthermore, in order to investigate time trends, the year of nursing home admission was used to construct five two-year cohorts (2011–2012, 2013–2014, 2015–2016, 2017–2018, and 2019–2020).

To assess survival times after nursing home admission, information on date of nursing home admission and duration of nursing home placement were obtained from statutory LTC data, which includes information on both outpatient and inpatient nursing care (Blümel et al. 2020). Information on care dependency was also obtained from statutory LTC insurance. In light of the findings of previous analyses, the old levels and new grades were combined into three groups of care need, as follows: low (care level: 1; grade: 1/2), medium (care level: 2; grade: 3/4), and high (care level: 3; grade: 5) (Schnakenberg et al. 2022).

Furthermore, data on confirmed outpatient diagnosis were used to identify the prevalence of dementia and cancer. These diagnoses were coded according the International Classification of Diseases, 10th Revision, German Modification (ICD-10-GM). Dementia was assessed using ICD-10-GM codes F00.x, F01.x, F02.0, F02.3, F03, G30.x, G31.0, G31.1, G31.82, G31.9 (Czwikla et al. 2023) and cancer using C00-D48 (Schnakenberg et al. 2022). At least one of the respective codes had to be present in the last three quarters before or in the quarter of nursing home admission.

### Statistical analysis

The study population was analysed descriptively, stratified by the 5 two-year cohorts of nursing home admission. For categorical variables proportions were used. For continuous variables, mean values with standard deviations (SDs) as well as medians with interquartile ranges (IQRs) were calculated. To assess sex, age, care need, dementia diagnosis, cancer diagnosis, and time trend differences in survival times after nursing home admission (10%, 25%, and 50% deceased), the Kaplan–Meier method with 95% Confidence Intervals (CI) of Hall-Wellner was used (Kaplan and Meier 1958). Observation time started with the day of nursing home admission and ended at the day of death, end of the insurance period or end of follow-up (December 31, 2021), whichever came first. Additionally, proportions of NHRs being deceased within 7, 30, 90, 180, and 365 days after nursing home admission were calculated with corresponding 95% CIs. In order to investigate differences in residents with dementia and cancer diagnosis, survival times and mortality were also calculated in those populations.

A Cox proportional hazard model was applied to determine factors associated with mortality. Hazard ratio (HR) with 95% CI were estimated with non-overlapping 95% CIs being considered statistically significant. First, we performed univariable analyses, including year of nursing home admission (2011–2012, 2013–2014, 2015–2016, 2017–2018, 2019–2020), sex (female, male), age (65–74, 75–84, 85–94, 95 + years), care need (low, medium, high), dementia diagnosis (yes, no), and cancer diagnosis (yes, no) as independent variables. Finally, all variables were included in a multivariable model.

All analyses were conducted using SAS 9.4 (SAS Institute Inc, Cary, North Carolina, United States).

## Results

### Baseline characteristics of the study population

A total of 113,829 residents met the inclusion criteria and were newly admitted to a nursing home between 2011 and 2020. These residents were on average 83.8 years old, 69% were female and 49% had a medium or high care need (Table 1). Almost half (48%) had a diagnosis of dementia and 34% a cancer diagnosis at the time of institutionalization. Furthermore, 30% of residents with dementia had cancer, and 42% of residents with cancer had dementia (Supplementary material 1 and Supplementary material 2).

**Table 1:** Characteristics of the study population stratified by the year of nursing home admission

Characteristics	2011/2012 N = 21,825	2013/2014 N = 23,828	2015/2016 N = 23,265	2017/2018 N = 22,812	2019/2020 N = 22,099	TOTAL N = 113,829
*Sex (n* = *113,829)*						
Male	29.2%	29.8%	31.2%	32.0%	32.6%	31.0%
Female	70.8%	70.2%	68.8%	68.0%	67.4%	69.0%
*Age in years (n* = *113,829)*						
Mean ± SD	83.5 ± 7.0	83.6 ± 7.0	83.8 ± 7.0	84.0 ± 7.1	84.1 ± 7.1	83.8 ± 7.0
Median [IQR]	84.0 [79.0–89.0]	84.0 [79.0–89.0]	84.0 [79.0–89.0]	85.0 [80.0–89.0]	85.0 [80.0–89.0]	84.0 [79.0–89.0]
65–74	12.2%	11.2%	10.4%	10.2%	10.9%	11.0%
75–84	40.1%	40.4%	40.0%	39.5%	38.1%	39.6%
85–94	43.3%	44.7%	45.0%	45.0%	45.6%	44.7%
95 +	4.5%	3.7%	4.6%	5.3%	5.4%	4.7%
*Care need (n* = *113,829)*						
Low (care level: 1, grade: 1/2)	59.4%	58.3%	54.7%	37.4%	47.4%	51.5%
Medium (care level: 2, grade: 3/4)	33.5%	33.4%	36.1%	55.2%	45.9%	40.8%
High (level: 3, grade: 5)	7.1%	8.4%	9.2%	7.4%	6.7%	7.8%
*Diagnosis (n* = *113,829)*						
Dementia, yes	45.2%	47.0%	48.5%	50.0%	48.2%	47.8%
Cancer, yes	30.3%	33.1%	34.4%	35.6%	37.0%	34.1%

SD = Standard Deviation; IQR = Inter Quartile Range

Regarding time trends, proportion of males and the mean age at nursing home admission increased from 29% and 83.5 years in 2011/2012 to 33% and 84.1 years in 2019/2020, respectively. The percentage of NHRs with low care needs ranged from 55 to 59% between 2011/2012 and 2015/2016, decreased to 37% in 2017/2018, and then rose to 47% in 2019/2020. The prevalence of dementia diagnosis remained nearly unchanged (45% to 48%) from 2011/2012 to 2019/2020 while the prevalence of cancer raised from 30 to 37%.

### Survival time and mortality after nursing home admission

**Fig. 1:**
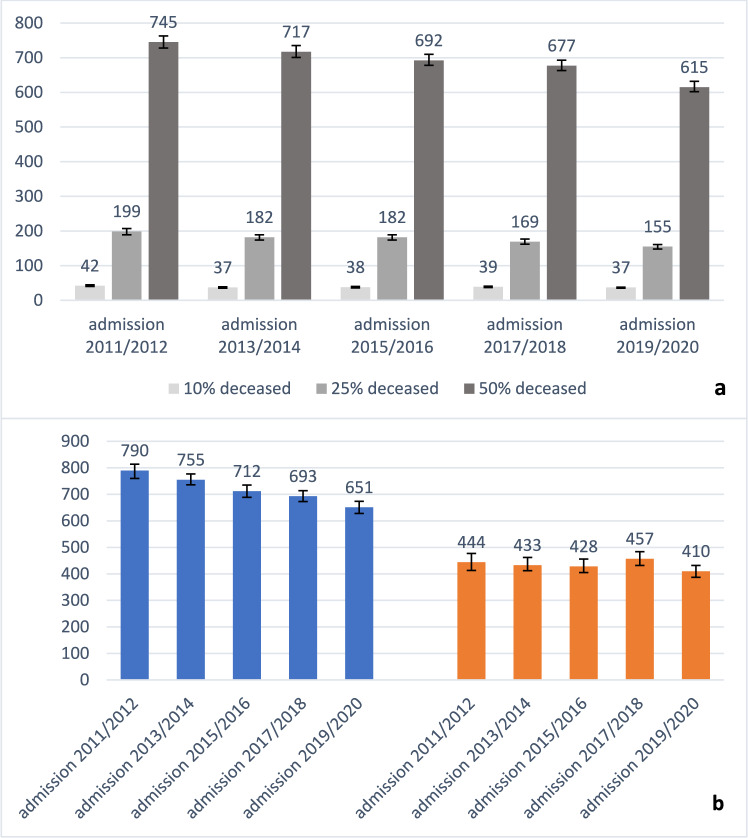
**a** Proportion of deceased nursing home residents (10%, 25% and 50%) in days with 95% confidence limits by year of nursing home admission; **b** Median survival time (50% deceased) in days with 95% confidence limits in those with dementia (blue) and cancer (orange) diagnosis and their year of nursing home admission

Overall, 10% of the residents died within 38 days (95% CI 38–39) and 25% within 176 days (95% CI 173–180) after nursing home admission (see Table 2 and Fig. 1). The median survival time was 690 days (95% CI 683–698). Regarding mortality, 2% died within 7 days, 8% within 30 days, and over one-third within the first year after nursing home admission.

**Table 2:** Survival times and mortality by sex, age, care need, dementia and cancer diagnosis as well as year of nursing home admission

Characteristic	10% deceased in days (95% CI)	25% deceased in days (95% CI)	50% deceased in days (95% CI)	% deceased within 7 days (95% CI)	% deceased within 30 days (95% CI)	% deceased within 90 days (95% CI)	% deceased within 180 days (95% CI)	% deceased within 365 days (95% CI)
Overall (n = 113,829)	38 (38–39)	176 (173–180)	690 (683–698)	2.3 (2.2–2.4)	8.4 (8.2–8.5)	17.4 (17.2–17.6)	25.0 (24.7–25.3)	35.4 (35.1–35.7)
*Sex (n* = *113,829)*								
Male	26 (25–27)	111 (108–115)	480 (469–492)	3.2 (3.0–3.4)	11.1 (10.8–11.4)	22.2 (21.8–22.7)	31.4 (30.9–31.9)	43.5 (43.1–44.1)
Female	47 (46–48)	224 (218–230)	800 (790–811)	1.9 (1.8–2.0)	7.1 (6.9–7.3)	15.3 (15.0–15.5)	22.1 (21.8–22.4)	31.7 (31.4–32.0)
*Age in years (n* = *113,829)*								
65–74	24 (22–26)	139 (129–150)	819 (777–851)	3.8 (3.5–4.2)	11.4 (10.9–12.0)	20.6 (19.9–21.3)	27.2 (26.5–28.0)	35.5 (34.6–36.3)
75–84	39 (38–41)	192 (185–199)	786 (773–802)	2.3 (2.2–2.5)	8.3 (8.0–8.5)	16.7 (16.3–17.0)	23.9 (23.5.24.3)	33.3 (32.9–33.7)
85–94	43 (41–44)	183 (177–187)	647 (637–656)	1.8 (1.7–1.9)	7.5 (7.3–7.7)	16.8 (16.5–17.2)	24.7 (24.3–25.0)	36.1 (35.7–36.5)
95 +	30 (27–32)	114 (104–123)	419 (402–445)	2.8 (2.4–3.3)	10.3 (9.5–11.1)	21.9 (20.8–23.0)	32.0 (30.7–33.2)	45.8 (44.5–47.2)
*Care need (n* = *113,829)*								
Low (care level: 1, grade: 1/2)	93 (89–96)	400 (391–409)	1097 (1084–1110)	1.3 (1.2–1.4)	4.3 (4.2–4.5)	9.7 (9.5–10.0)	15.0 (14.7–15.3)	23.0 (22.6–23.3)
Medium (care level: 2, grade: 3/4)	30 (28–31)	113 (110–116)	448 (438–456)	2.6 (2.4–2.7)	10.2 (10.0–10.5)	21.9 (21.5–22.3)	31.9 (31.4–32.3)	45.0 (44.5–45.4)
High (level: 3, grade: 5)	10 (9–11)	30 (28–32)	125 (117–134)	7.4 (6.9–8.0)	25.1 (24.2–26.0)	44.7 (43.6–45.7)	55.1 (54.1–56.2)	67.0 (66.0–68-0)
*Dementia diagnosis (n* = *113,829)*								
Yes	55 (53–57)	214 (210–220)	719 (709–730)	1.5 (1.4–1.6)	6.1 (5.9–6.3)	14.6 (14.3–14.9)	22.4 (22.0–22.7)	33.4 (33.1–33.8)
No	28 (28–30)	142 (138–147)	663 (652–673)	3.0 (2.9–3.2)	10.4 (10.2–10.7)	20.0 (19.7–20.4)	27.4 (27.1–27.8)	37.1 (36.7–37.5)
*Cancer diagnosis (n* = *113,829)*								
Yes	19 (19–20)	81 (79–84)	433 (422–445)	4.1 (3.9–4.2)	13.8 (13.5–14.1)	26.3 (25.8–26.7)	35.2 (34.7–35.6)	46.1 (45.6–46.6)
No	63 (61–65)	263 (257–268)	825 (816–836)	1.4 (1.3–1.5)	5.5 (5.4–5.7)	12.8 (12.6–13.1)	19.7 (19.5–20.0)	29.8 (29.5–30.2)
*Year of nursing home admission (n* = *113,829)*								
2011/2012	42 (40–45)	199 (189–207)	745 (728–763)	2.2 (2.0–2.4)	7.9 (7.5–8.3)	16.4 (15.9–16.9)	23.7 (23.1–24.2)	34.1 (33.4–34-7)
2013/2014	37 (35–39)	182 (174–189)	717 (701–735)	2.7 (2.5–2.9)	8.6 (8.2–8.9)	17.5 (17.0–18.0)	24.8 (24.2–25.3)	34.8 (34.8–35-4)
2015/2016	38 (36–40)	182 (174–189)	692 (678–710)	2.4 (2.2–2.6)	8.4 (8.0–8.7)	17.3 (16.8–17.8)	24.6 (24.1–25.2)	35.4 (34.8–36.1)
2017/2018	39 (37–41)	169 (162–177)	677 (663–693)	2.1 (1.9–2.2)	8.3 (8.0.8.7)	17.4 (17.0–17.9)	25.5 (24.9–26.0)	35.8 (35.2–36.4)
2019/2020	37 (35–38)	155 (148–161)	615 (602–632)	2.1 (1.9–2.3)	8.6 (8.2–8.9)	18.5 (18.0–19.0)	26.4 (25.9–27.0)	36.8 (36.1–37.4)

CI = Confidence Interval

Overall, times during which 25% of residents died decreased from 199 days (95% CI 189–207) in 2011/2012 to 155 days (95% CI 148–161) in 2019/2020 (Table 2). Median survival time changed from 745 days (95% CI 728–763) to 615 days (95% CI 602–632) (see also Supplementary material 3). Except for the early mortality within 7 days, the relative difference in mortality between the two-year cohorts is quite similar for all time frames. This results in an increased mortality within 365 days after institutionalization from 34% in 2011/2012 to 37% in 2019/2020.

#### Survival and mortality focusing on dementia and cancer

Compared to women, shorter survival times and higher mortality were observed in men in every period after nursing home admission. The same holds true when comparing higher to lower care need. Higher survival times and lower mortality was observed in individuals with dementia compared to individuals without dementia diagnosis (median survival: 719 days vs. 663 days). On the contrary, median survival time in residents with cancer was shorter (433 days) compared to those without (825 days).

Focusing on time trends in residents with dementia and cancer diagnosis respectively, survival times decreased continuously from 2011/2012 to 2019/2020 in residents with dementia (Table 3). This pattern was observed right after admission to nursing home and resulted in a median survival time of 790 days (95% CI 760–814) in 2011/2012 and 651 days (95% CI 628–674) in 2019/2020. This trend was also found in residents without dementia (Supplementary material 4). Conversely, survival times and mortality remained nearly unchanged in cancer patients.

**Table 3:** Survival times and mortality in residents with cancer and dementia diagnosis

Characteristic	10% deceased in days (95% CI)	25% deceased in days (95% CI)	50% deceased in days (95% CI)	% deceased within 7 days (95% CI)	% deceased within 30 days (95% CI)	% deceased within 90 days (95% CI)	% deceased within 180 days (95% CI)	% deceased within 365 days (95% CI)
Dementia (n = 54,392)								
Overall	55 (53–57)	214 (210–220)	719 (709–730)	1.5 (1.4–1.6)	6.1 (5.9–6.3)	14.6 (14.3–14.9)	22.4 (22.0–22.79	33.4 (33.1–33.8)
*Year of nursing home admission*								
2011/2012	62 (59–67)	243 (232–260)	790 (760–814)	1.3 (1.0–1.5)	5.7 (5.2–6.1)	13.4 (12.8–14.1)	20.6 (19.8–21.4)	31.7 (30.8–32.6)
2013/2014	59 (55–63)	241 (225–252)	755 (736–777)	1.7 (1.5–2.0)	6.0 (5.5–6.4)	13.9 (13.3–14.5)	21.1 (20.4–22.0)	31.8 (31.0–32-7)
2015/2016	55 (52–59)	221 (210–232)	712 (689–735)	1.5 (1.3–1.7)	6.0 (5.6–6.5)	14.4 (13.8–15.1)	22.0 (21.2–22.7)	33.7 (32.8–34.5)
2017/2018	53 (48–57)	193 (184–203)	693 (673–714)	1.3 (1.1–1.5)	6.2 (5.8–6.7)	15.0 (14.3–15.6)	23.8 (23.0–24.6)	35.1 (34.2–36.0)
2019/2020	48 (45–52)	186 (178–197)	651 (628–674)	1.5 (1.3–1.7)	6.6 (6.1–7.0)	16.0 (15.3–16.7)	24.1 (23.3–24-9)	34.8 (33.9–35.7)
Cancer (n = 38,788)								
Overall	19 (19–20)	81 (79–84)	433 (423–445)	4.1 (3.9–4.2)	13.8 (13.5–14.1)	26.3 (25.8–26.7)	35.2 (34.7–35.6)	46.1 (45.6–46.6)
*Year of nursing home admission*								
2011/2012	18 (17–20)	82 (76–88)	444 (413–477)	4.4 (3.9–4.8)	13.8 (13.0–14.7)	26.3 (25.3–27.4)	35.2 (34.1–36.4)	46.1 (44.9–47.3)
2013/2014	17 (15–19)	76 (70–81)	433 (412–462)	4.8 (4.4–5-3)	14.6 (13.8–15.4)	27.3 (26.3–28.2)	36.0 (34.9–37.0)	46.6 (45.5–47.7)
2015/2016	19 (17–21)	81 (75–87)	428 (405–456)	4.4 (4.0–4.9)	14.2 (13.4–15.0)	26.4 (25.4–27.3)	34.5 (33.5–35.6)	46.3 (45.3–47.4)
2017/2018	21 (20–23)	88 (81–95)	457 (432–484)	3.3 (2.9–3.7)	13.1 (12.4–13.9)	25.1 (24.2–26.1)	34.4 (33.4–35.4)	45.0 (43.9–46.1)
2019/2020	21 (19–22)	81 (76–86)	410 (387–432)	3.5 (3.1–3-9)	13.3 (12.6–14.0)	26.3 (25.4–27.3)	35.7 (34.7–36.7)	46.4 (45.4–47.5)

CI = Confidence Interval

### Factors associated with mortality: results from crude and multivariable cox proportional hazard model

Crude cox proportional hazard models showed that—compared to the years 2011/2012—later years of nursing home admission were significantly associated with mortality (Table 4) with HRs increasing from 1.03 (95% CI 1.01–1.05) in 2013/2014 to 1.16 (95% CI 1.14–1.19) in 2019/2020. Furthermore, crude models also showed the influence on mortality of male sex (HR: 1.37; 95% CI 1.35–1.39), increasing age, higher care need, and having a cancer diagnosis (HR: 1.44; 95% CI 1.42–1.45), while having a dementia diagnosis had no influence.

**Table 4:** Cox regression models for factors associated with mortality (n = 113,829)

Characteristic		Crude model	Multivariable model
	Reference	HR (95% CI)	
*Sex*			
Male	Female	1.37 (1.35–1.39)	1.40 (1.38–1.42)
*Age in years*			
75–84	65–74	1.10 (1.08–1.13)	1.19 (1.16–1.23)
85–94	65–74	1.36 (1.32–1.39)	1.54 (1.51–1.58)
95 +	65–74	1.88 (1.81–1.94)	2.08 (2.00–2.15)
*Care need*			
Medium (care level: 2, grade: 3/4)	Low (care level: 1, grade: 1/2)	1.88 (1.85–1.91)	1.89 (1.87–1.92)
High (level: 3, grade: 5)	Low (care level: 1, grade: 1/2)	3.15 (3.08–3.22)	3.28 (3.20–3.36)
*Dementia diagnosis*			
Yes	No	0.99 (0.98–1.01)	0.90 (0.89–0.92)
*Cancer diagnosis*			
Yes	No	1.44 (1.42–1.45)	1.39 (1.37–1.41)
*Year of nursing home admission*			
2013/2014	2011/2012	1.03 (1.01–1.05)	1.02 (1.00–1.04)
2015/2016	2011/2012	1.07 (1.05–1.09)	1.01 (0.99–1.03)
2017/2018	2011/2012	1.10 (1.08–1.13)	0.96 (0.94–0.98)
2019/2020	2011/2012	1.16 (1.14–1.19)	1.09 (1.07–1.12)

HR = Hazard Ratio; CI = Confidence Interval

In the multivariable model, no clear time trends were observed. Nevertheless, admission to nursing home in the years 2019/2020 compared to 2011/2012 was associated with higher mortality (HR: 1.09; 95% CI 1.07–1.12).

## Discussion

Between 2011/2012 and 2019/2020, we found an increasing mean age, rising proportions of men, and a higher prevalence of cancer diagnoses among newly admitted NHRs, while proportions with dementia remained almost stable. Regarding the whole nursing home cohort, survival times decreased and mortality increased during this period. This development was driven by residents who had no cancer diagnosis, while survival times in cancer patients remained nearly unchanged.

### Trends in characteristics of nursing home residents

Regarding sex and age, we found increasing proportions of men (from 29 to 33%) and a slightly rise of the mean age from 83.5 to 84.1 years between 2011/2012 and 2019/2020. Data from the ELSA project confirms these findings, although to a greater extent (proportion of men from 33 to 42%; mean age from 81.0 to 85.0 years). The differences between the studies might be explained by the small sample size of the ELSA study and the earlier observation period (2002/2003 to 2014/2015) (Green et al. 2017). However, the ELSA study is situated in a different country context, where the mechanisms and timing of nursing home admission may differ significantly.

Our observed prevalence of dementia (45%-48%) is in line with many previous findings across countries (Hoffmann et al. 2014; Xu et al. 2016; Allers and Hoffmann 2018). However, studies on dementia trends over time of newly admitted NHR barely exist. An analysis from nursing homes in the UK, Australia, New Zealand and Spain suggests an increase of dementia from 36% in 2003 to 44% in 2009 (Lievesley et al. 2011). We also found an overall prevalence of cancer of 34%, which is higher than the prevalence in NHRs found in studies from France (8%) (Liuu et al. 2019), the US (10–15%) (Rodin 2008), or Denmark (25%) (Reilev et al. 2019). However, comparisons are hampered due to different health care systems, definitions/inclusion of different cancer sites, or populations. Regarding time trends, we found a clear increase of cancer prevalence from 30% in 2011/2012 to 37% in 2019/2020, which is in line with the ELSA study, that also found a rising trend (Green et al. 2017). These results might suggest improved survival rates for older cancer patients, and as both the prevalence of cancer and care dependency increases dramatically with age (Yancik and Ries 2000; Marengoni et al. 2011; European Commission 2021) they mirror demographic changes.

### Decreasing survival

We found an overall median survival time of 690 days and a mortality within 12 months after institutionalization of 35%. These results that the first months after admission to a nursing home represent a period at high risk of mortality are confirmed by several previous studies (Hedinger et al. 2015; Vetrano et al. 2018; Allers and Hoffmann 2018; Braggion et al. 2020), although the majority of studies found higher survival times (Dale et al. 2001; McCann et al. 2009; Wieland et al. 2010; Hjaltadáttir et al. 2011; Li et al. 2018; Vossius et al. 2018) and lower mortality (Hjaltadáttir et al. 2011; Tabue-Teguo et al. 2015; Vossius et al. 2018; Reilev et al. 2019; Collingridge Moore et al. 2020). Furthermore, our observed short-term mortality after institutionalisation appears higher than in some other studies, though cross-country comparisons are challenging, as survival in nursing homes largely depends on whether and when individuals enter such care. These patterns may vary across countries due to differences in LTC systems and societal norms regarding institutionalisation. In a study from Australia, Jorisson et al. showed a mortality of 9% within 90 days after entering a nursing home, which is almost half of ours (17%) (Jorissen et al. 2024). A Scottish study (MacRae et al. 2024) also reported lower mortality at 30 days (3%) and 180 days (15%) after admission compared to our findings (8% and 25%). However, mortality among nearly 20,000 newly admitted NHRs in Italy (Braggion et al. 2020) at 6 and 12 months was nearly identical to ours (23% and 34% vs. 25% and 35%). Overall, more studies investigating short-term mortality after nursing home admission including international comparisons are needed. Over the decade, a major finding of this study is the decline in survival times among NHRs. Median survival decreased from 745 days in 2011/2012 to 615 days in 2019/2020. This decline in survival echoes findings from Sweden (Schön et al. 2016) and from two of three studied Canadian regions (Hoben et al. 2019), suggesting that nursing homes increasingly care for older, frailer residents with complex health needs and higher levels of care dependency. The increasing proportion of men being admitted to nursing homes (with shorter survival times compared to women) may also contribute to higher overall mortality.

Although our prevalence of dementia remained nearly stable, survival times among residents with dementia consistently declined over the years with the median survival time decreasing from 790 days in 2011/2012 to 651 days in 2019/2020. A similar pattern was also found in residents without dementia. Furthermore, our finding of increased cancer prevalence also contributed substantially to the declining survival times and increasing mortality over the years. In line with a recent review (Moore et al. 2019), our results show that residents with cancer had much shorter survival times, with half dying within 433 days compared to 825 days for those without cancer, highlighting the considerable burden of cancer in this population. However, depending on the cancer entity and the progression of the disease, survival times, symptoms and the required assistance can vary greatly. For example, an analysis from the US reported that 38% of cancer patients newly admitted to nursing homes were experiencing no pain and only 5% were receiving chemotherapy or radiation (Buchanan et al. 2005). This suggests a significant group of patients with cancer being admitted to nursing homes who are not necessarily at the end of life (Rodin 2008). For these patients, moving to a nursing home seems to be reasonable. On the other hand, the study from the US also showed that 21% of newly admitted NHRs were judged to be in their final 6 months of life, corresponding to our finding that one quarter of residents with cancer had died 81 days after admission. This suggest that moving to a nursing home for those individuals might be very stressful. Furthermore, studies have shown that these residents often receive inadequate palliative care in nursing homes, but instead aggressive end of life care (Teno et al. 2004; Koroukian et al. 2023).

Our results of shorter survival times suggest that a more vulnerable population is being admitted to nursing homes. This indicates that even those with substantial care needs are more often cared for at home, underscoring the growing need to expand access to specialised outpatient and palliative care services (SAPV). Although SAPV have been introduced in Germany over the past decades (Schneider et al. 2018), there remains a substantial unmet need for palliative care, particularly in nursing homes. Utilisation of SAPV is low overall—with only 9.2% among eligible individuals in private households and just 4.7% among NHRs —highlighting significant gaps in service provision (Rehner et al. 2025). Studies further indicate that end of life care in nursing homes often fails to align with residents’ preferences (van Oorschot et al. 2019), and nursing home managers have identified poor access to general practitioners, insufficient staff training, and low SAPV enrollment as major barriers (Strautmann et al. 2020). In light of an increasing number of residents dying in these facilities, nursing homes must develop strategies to better meet these complex needs (Buchanan et al. 2002; Honinx et al. 2021). At the same time, ensuring adequate palliative care for older patients with cancer outside institutional settings remains a challenge. Although national efforts have expanded hospice infrastructure—now comprising around 1500 outpatient and 260 inpatient hospices (Deutscher Hospiz-und Palliativverband 2024)—these services are still predominantly geared towards younger cancer patients, with older individuals rarely receiving hospice care (Virnig et al. 2000; Lackan et al. 2004; O’Connor et al. 2014; Ditscheid et al. 2023). Access is further limited in rural areas due to fragmented service coordination and a lack of awareness among general practitioners (Sophie et al. 2020; Ditscheid et al. 2020, 2023). Nevertheless, there seems to be a clear need for expanding of such services both within and outside nursing homes.

### Strengths and limitations

A major strength of this study is the large cohort with a sample size of over 113,000 newly admitted NHRs, allowing for robust analyses of time trends over a 10-year period for this vulnerable population. In addition, this is the first trend analysis in characteristics and survival times of NHRs in Germany.

Nevertheless, some limitations must be considered. We did not have information on socioeconomic factors (e.g., marital status, house ownership, education), which are known to influence the length of nursing home stay (Hedinger et al. 2015). Moreover, information on clinical characteristics or the disease severity of dementia and cancer patients (e.g., cognitive or functional impairments, activities of daily living, pain, tumor stage) were not available in German health insurance data. However, information on care levels and care grades as indicator of cognitive, functional or psychological impairments were accessible. Though, with modifying care levels to care grades in 2017 (Nadash et al. 2018), our observed proportions of German NHRs with low and medium care need have changed substantially. In this context, the high proportion of nursing home admissions with low care needs may in part reflect the limitations of the former care level system (until 2016), which undervalued support needs related to cognitive impairment. This may have resulted in persons with substantial supervision needs but limited physical impairment being categorized as low care level, thereby underestimating the complexity of care required upon admission. Moreover, the subsequent shift in 2019/20 may reflect a stabilization or “recalibration” of the assessment practices, as care assessors and institutions adjusted to the new criteria, rather than actual changes in the care needs of new nursing home entrants. Therefore, these findings on trends should be interpreted with caution. In addition, we assumed that residents remained in the nursing homes without being discharged home. However, given that the proportion of residents discharged home is expected to be minimal, it is unlikely to significantly affect our results. Furthermore, the representativeness of the data needs to be discussed, as German insurance funds differ with respect to, e.g., sex, age, socioeconomic factors and morbidity (Hoffmann and Icks 2012). Although the population insured with AOK Niedersachsen does not differ from the general German population in terms of sex distribution, it includes a higher proportion of individuals with a low socioeconomic status (Epping et al. 2021), which—given the substantial out-of-pocket costs for nursing home care in Germany—may result in delayed admissions and thus contribute to an overestimation of short lengths of stay in our study. However, this is unlikely to affect our time trends. Lastly, our observation period included the COVID-19-pandemic, which could have influenced survival times and mortality. However, when further analyzing mortality between months and quarters of the study years, no differences in 2020 were found suggesting that the COVID-19-pandemic did not hamper our findings.

## Conclusions

This study found an increase in the proportion of patients with cancer newly admitted to nursing homes between 2011 and 2020. At the same time, the survival times decreased in the overall population as well as in residents with dementia, but not in those with cancer, who already experience higher mortality. The findings emphasize the necessity of integrating palliative care in nursing homes to support a frailer and increasingly vulnerable resident population being more and more at the end of life. Furthermore, appropriate care for older patients with cancer outside nursing homes need to be provided. In addition, more studies should assess time trends on changing characteristics and survival of NHRs also focusing on the important subgroups with dementia and cancer.

## Supplementary Information

Below is the link to the electronic supplementary material.

Supplementary file1 (DOCX 302 kb)

## Data Availability

No datasets were generated or analysed during the current study.
