# Supplementary material for: Change over time in characteristics and survival of residents newly admitted to nursing homes: an analysis of health insurance claims data from 2011 to 2020 in Germany
Source: Eur J Ageing. 2025 Jul 21;22(1):34. doi: 10.1007/s10433-025-00871-z (PMC12279626; doi:10.1007/s10433-025-00871-z)
Supplement: Supplementary file 1 — Supplementary file1 (DOCX 302 kb) [file 10433_2025_871_MOESM1_ESM.docx]

# Supplementary material

Supplementary material 1: Characteristics of residents with dementia diagnosis stratified by the year of nursing home admission

| **Characteristics** | **2011/2012**  **N=9,868** | **2013/2014**  **N=11,205** | **2015/2016**  **N=11,274** | **2017/2018**  **N=11,395** | **2019/2020**  **N=10,650** | **TOTAL**  **N=54,392** |
| --- | --- | --- | --- | --- | --- | --- |
| **Sex (n=54,392)** |  |  |  |  |  |  |
| Male | 28.8% | 29.2% | 31.0% | 32.2% | 32.4% | 30.7% |
| Female | 71.3% | 70.8% | 69.0% | 67.8% | 67.6% | 69.3% |
| **Age in years, (n=54,392)** |  |  |  |  |  |  |
| Mean ± SD | 84.0 (6.5) | 84.1 (6.5) | 84.2 (6.5) | 84.5 (6.5) | 84.6 (6.5) | 84.3 (6.5) |
| Median [IQR] | 84.0  [80.0-89.0] | 85.0  [80.0-89.0] | 85.0  [80.0-89.0] | 85.0  [80.0-89.0] | 85.0  [81.0-89.0] | 85.0  [80.0-89.0] |
| 65-74 | 8.7% | 8.1% | 7.2% | 6.7% | 7.3% | 7.6% |
| 75-84 | 42.1% | 41.9% | 41.5% | 41.6% | 40.0% | 41.4% |
| 85-94 | 44.8% | 46.4% | 46.8% | 46.5% | 47.5% | 46.4% |
| 95+ | 4.4% | 3.7% | 4.6% | 5.2% | 5.3% | 4.6% |
| **Care need, (n=54,392)** |  |  |  |  |  |  |
| Low (care level: 1, grade: 1/2) | 54.7% | 53.7% | 49.7% | 28.1% | 40.9% | 45.2% |
| Medium (care level: 2, grade: 3/4) | 36.4% | 36.2% | 39.4% | 63.1% | 51.8% | 45.6% |
| High (level: 3, grade: 5) | 8.9% | 10.1% | 10.9% | 8.7% | 7.3% | 9.2% |
| **Diagnosis (n=54,392)** |  |  |  |  |  |  |
| Cancer, yes | 26.3% | 28.6% | 30.3% | 31.7% | 33.5% | 30.1% |

SD=Standard Deviation; IQR=Inter Quartile Range

Supplementary material 2:Characteristics of residents with cancer diagnosis stratified by the year of nursing home admission

| **Characteristics** | **2011/2012**  **N=6,620** | **2013/2014**  **N=7,886** | **2015/2016**  **N=7,993** | **2017/2018**  **N=8,120** | **2019/2020**  **N=8,169** | **TOTAL**  **N=38,788** |
| --- | --- | --- | --- | --- | --- | --- |
| **Sex (n=38,788)** |  |  |  |  |  |  |
| Male | 37.3% | 37.5% | 39.4% | 39.7% | 39.9% | 38.8% |
| Female | 62.7% | 62.5% | 60.6% | 60.3% | 60.1% | 61.2% |
| **Age in years, (n=38,788)** |  |  |  |  |  |  |
| Mean ± SD | 82.9 (7.1) | 83.1 (6.9) | 83.2 (6.9) | 83.7 (7.0) | 83.8 (7.1) | 83.4 (7.0) |
| Median [IQR] | 83.0  [78.0-88.0] | 84.0  [78.0-88.0] | 84.0  [79.0-88.0] | 84.0  [79.0-89.0] | 84.0  [80.0-89.0] | 84.0  [79.0-88.0] |
| 65-74 | 14.2% | 12.1% | 11.3% | 10.7% | 11.5% | 11.9% |
| 75-84 | 41.4% | 42.5% | 41.9% | 41.2% | 39.7% | 41.3% |
| 85-94 | 40.4% | 42.5% | 43.2% | 43.6% | 43.9% | 42.8% |
| 95+ | 4.0% | 2.9% | 3.6% | 4.6% | 5.0% | 4.0% |
| **Care need, (n=38,788)** |  |  |  |  |  |  |
| Low (care level: 1, grade: 1/2) | 58.4% | 57.0% | 53.1% | 35.6% | 43.1% | 49.0% |
| Medium (care level: 2, grade: 3/4) | 34.4% | 33.0% | 36.3% | 56.0% | 48.7% | 42.0% |
| High (level: 3, grade: 5) | 7.2% | 9.9% | 10.6% | 8.5% | 8.2% | 8.9% |
| **Diagnosis (n=38,788)** |  |  |  |  |  |  |
| Dementia, yes | 39.2% | 40.6% | 42.7% | 44.5% | 43.6% | 42.2% |

SD=Standard Deviation; IQR=Inter Quartile Range

Supplementary material 3: Kaplan–Meier-Plot: Survival probability of nursing home residents in the years 2011/2012 (n=21,825) and 2019/2020 (n=22,099) with 95% Hall–Wellner Bands and number of individuals at risk


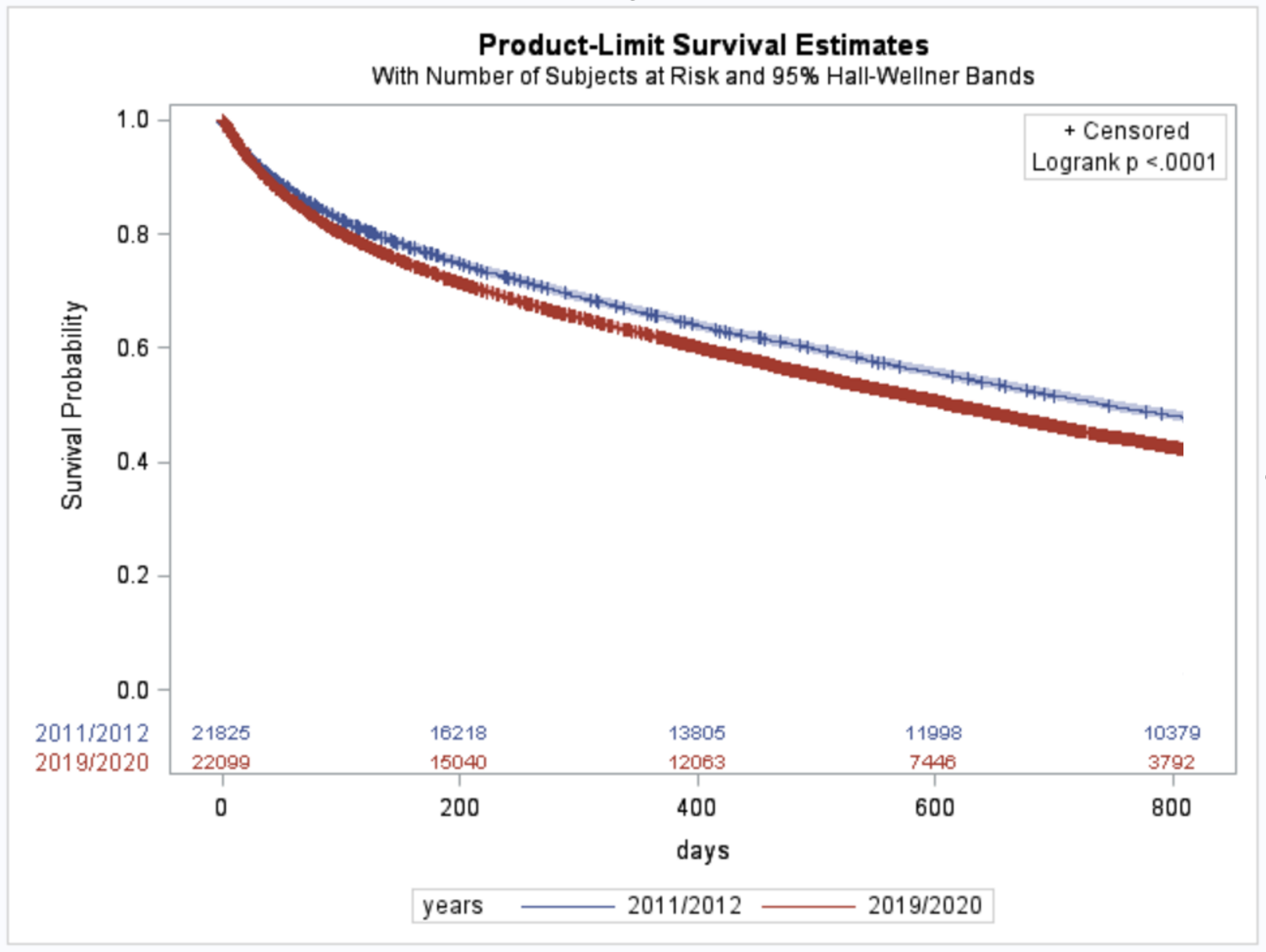


Supplementary material 4: Survival times and mortality in residents without dementia and cancer diagnosis

| **Characteristic** | **10% deceased in days (95% CI)** | **25% deceased in days (95% CI)** | **50% deceased in days (95% CI)** | **% deceased within 7 days (95% CI)** | **% deceased within 30 days (95% CI)** | **% deceased within 90 days (95% CI)** | **% deceased within 180 days (95% CI)** | **% deceased within 365 days (95% CI)** |
| --- | --- | --- | --- | --- | --- | --- | --- | --- |
| **Without dementia (n=59,437)** |  |  |  |  |  |  |  |  |
| **Overall** | 28 (28-30) | 142 (138-147) | 663 (652-673) | 3.0 (2.9-3.2) | 10.4 (10.2-10.7) | 20.0 (19.7-20.4) | 27.4 (27.1-27.8) | 37.1 (36.7-37.5) |
| **Year of nursing home admission** |  |  |  |  |  |  |  |  |
| 2011/2012 | 32 (29-35) | 162 (151-172) | 711 (689-735) | 2.9 (2.6-3.2) | 9.7 (9.2-10.3) | 18.8 (18.1-19.5) | 26.2 (25.4-27.0) | 36.0 (35.2-36.9) |
| 2013/2014 | 27 (25-29) | 139 (128-149) | 678 (654-702) | 3.5 (3.2-3.9) | 10.9 (10.3-11.4) | 20.6 (19.9-21.4) | 28.0 (27.2-28.7) | 37.4 (36.6-38.3) |
| 2015/2016 | 28 (26-30) | 145 (135-157) | 674 (651-697) | 3.2 (2.9-3.5) | 10.6 (10.0-11.1) | 20.0 (19.3-20.7) | 27.2 (26.4-28.0) | 37.1 (36.3-38.0) |
| 2017/2018 | 29 (27-31) | 145 (133-157) | 661 (637-684) | 2.8 (2.5-3.1) | 10.4 (9.9-11.0) | 19.9 (19.2-20.6) | 27.2 (26.3-28.0) | 36.5 (35.6-37.4) |
| 2019/2020 | 28 (27-31) | 125 (117-134) | 580 (555-603) | 2.7 (2.4-3.0) | 10.4 (9.8-11.0) | 20.8 (20.1-21.6) | 28.6 (27.8-29.5) | 38.6 (37.7-39.5) |
|  |  |  |  |  |  |  |  |  |
| **Without cancer (n=75,041)** |  |  |  |  |  |  |  |  |
| **Overall** | 63 (61-65) | 263 (257-268) | 825 (816-836) | 1.4 (1.3-1.5) | 5.5 (5.4-5.7) | 12.8 (12.6-13.1) | 19.7 (19.5-20.0) | 29.8 (29.5-30.2) |
| **Year of nursing home admission** |  |  |  |  |  |  |  |  |
| 2011/2012 | 68 (63-73) | 285 (275-297) | 879 (858-900) | 1.2 (1.0-1.4) | 5.3 (5.0-5.7) | 12.1 (11.5-12.6) | 18.6 (18.0-19.3) | 28.8 (28.1-29.5) |
| 2013/2014 | 65 (60-70) | 276 (264-291) | 853 (832-878) | 1.6 (1.4-1.8) | 5.6 (5.2-6.0) | 12.6 (12.1-13.1) | 19.2 (18.6-19.8) | 28.9 (28.2-29.6) |
| 2015/2016 | 66 (61-70) | 267 (256-278) | 837 (817-860) | 1.3 (1.1-1.5) | 5.3 (5.0-5.7) | 12.6 (12.1-13.1) | 19.5 (18.8-20.1) | 29.7 (29.0-30.5) |
| 2017/2018 | 61 (57-65) | 248 (235-262) | 795 (777-817) | 1.4 (1.2-1.6) | 5.7 (5.3-6.1) | 13.2 (12.6-13.7) | 20.5 (19.9-21.2) | 30.7 (30.0-31.5) |
| 2019/2020 | 58 (54-62) | 233 (221-244) | 737 (716-764) | 1.3 (1.1-1.5) | 5.8 (5.4-6.2) | 13.9 (13.3-14.5) | 21.0 (20.3-21.7) | 31.1 (30.3-31.9) |

CI=Confidence interval
